# Enhanced Operational Characteristics Attained by Applying HfO_2_ as Passivation in AlGaN/GaN High-Electron-Mobility Transistors: A Simulation Study

**DOI:** 10.3390/mi14061101

**Published:** 2023-05-23

**Authors:** Jun-Hyeok Choi, Woo-Seok Kang, Dohyung Kim, Ji-Hun Kim, Jun-Ho Lee, Kyeong-Yong Kim, Byoung-Gue Min, Dong Min Kang, Hyun-Seok Kim

**Affiliations:** 1 Division of Electronics and Electrical Engineering, Dongguk University-Seoul, Seoul 04620, Republic of Korea; junhyeok6293@dgu.ac.kr (J.-H.C.); kws1117@dongguk.edu (W.-S.K.); ehgudakr@dongguk.edu (D.K.); kjsuk0105@dongguk.edu (J.-H.K.); steve1211@dgu.ac.kr (J.-H.L.); kky0213@dongguk.edu (K.-Y.K.); 2 Electronics and Telecommunications Research Institute, Daejeon 34129, Republic of Korea; minbg@etri.re.kr (B.-G.M.); kdm1597@etri.re.kr (D.M.K.)

**Keywords:** AlGaN/GaN, high-electron-mobility transistor, passivation, HfO_2_

## Abstract

This study investigates the operating characteristics of AlGaN/GaN high-electron-mobility transistors (HEMTs) by applying HfO_2_ as the passivation layer. Before analyzing HEMTs with various passivation structures, modeling parameters were derived from the measured data of fabricated HEMT with Si_3_N_4_ passivation to ensure the reliability of the simulation. Subsequently, we proposed new structures by dividing the single Si_3_N_4_ passivation into a bilayer (first and second) and applying HfO_2_ to the bilayer and first passivation layer only. Ultimately, we analyzed and compared the operational characteristics of the HEMTs considering the basic Si_3_N_4_, only HfO_2_, and HfO_2_/Si_3_N_4_ (hybrid) as passivation layers. The breakdown voltage of the AlGaN/GaN HEMT having only HfO_2_ passivation was improved by up to 19%, compared to the basic Si_3_N_4_ passivation structure, but the frequency characteristics deteriorated. In order to compensate for the degraded RF characteristics, we modified the second Si_3_N_4_ passivation thickness of the hybrid passivation structure from 150 nm to 450 nm. We confirmed that the hybrid passivation structure with 350-nm-thick second Si_3_N_4_ passivation not only improves the breakdown voltage by 15% but also secures RF performance. Consequently, Johnson’s figure-of-merit, which is commonly used to judge RF performance, was improved by up to 5% compared to the basic Si_3_N_4_ passivation structure.

## 1. Introduction

Generally, AlGaN/GaN high-electron-mobility transistors (HEMTs) are widely adopted in power electronics because of their outstanding electronic and material properties, such as high-critical electric field (~3.3 MV/cm) and wide energy bandgap (3.4 eV). Interestingly, these remarkable characteristics make GaN more practicable for high-power and high-frequency applications compared to other materials [1]. Hence, due to these material characteristics, AlGaN/GaN HEMTs exhibit high electron saturation velocity as well as high current density, high thermal reliability, and high breakdown voltage ($V_{BD}$) [2,3,4]. In addition, HEMTs based on the AlGaN/GaN heterostructure show admirable performances via a two-dimensional electron gas (2-DEG) in the channel generated by the spontaneous and piezoelectric polarization effects [5,6]. Nevertheless, to sufficiently satisfy the market requirements, GaN-based HEMTs require further research for high-voltage and high-frequency applications [7,8,9]. It has been demonstrated that the field-plate structures in GaN-based HEMTs are commonly used to increase the $V_{BD}$, resulting in operational stability and reliability. However, the frequency characteristics are degraded due to the increase in parasitic capacitances, such as the gate-to-source capacitance ($C_{gs}$) and gate-to-drain capacitance ($C_{gd}$) [10,11]. This clearly shows the advantages and disadvantages of applying field-plates in GaN-based HEMTs due to trade-off between DC and RF characteristics. Additionally, many studies are being conducted to improve the devices’ performance [12,13]. As an alternative to HEMTs with field-plate structure, we employed HfO_2_ as the passivation to enhance $V_{BD}$. Interestingly, HfO_2_ has a high dielectric constant (~25) and large bandgap energy (5.7 eV), both of which may be exploited to improve the devices’ performance in comparison with the basic GaN-based HEMTs with Si_3_N_4_ passivation [14]. Based on these material properties, it is anticipated that the leakage current and $V_{BD}$ characteristics can be improved. However, HfO_2_ passivation in HEMTs also produces additional parasitic capacitances, which may degrade their frequency characteristics. Thus, we suggested the additional structures to secure RF performance while applying HfO_2_ as a passivation layer.

In this article, we compare and analyze three different passivation structures which use the basic Si_3_N_4_, only HfO_2_, and HfO_2_/Si_3_N_4_ (hybrid), respectively, as passivation materials. Compared to the basic Si_3_N_4_ passivation structure, we confirmed that the $V_{BD}$ of the HfO_2_ passivation structure improved by approximately 18.8%, but its frequency characteristics were significantly degraded. Meanwhile, the hybrid passivation structure exhibited a slightly reduced $V_{BD}$, but its frequency characteristics were improved to approximately twice that of the HfO_2_ passivation structure. Thus, we optimized the second Si_3_N_4_ passivation thickness in the hybrid passivation structure to further increase its RF performance. Consequently, the various passivation structures in terms of $V_{BD}$, on-resistance ($R_{on}$), and cut-off frequency ($f_{T}$) were evaluated using the standard lateral figure-of-merit (LFOM) (=${V_{BD}}^{2}/R_{on}$) and Johnson’s figure-of-merit (JFOM) (=$f_{T}\times V_{BD}$) [15,16,17].

## 2. Materials and Methods

**Figure 1:**
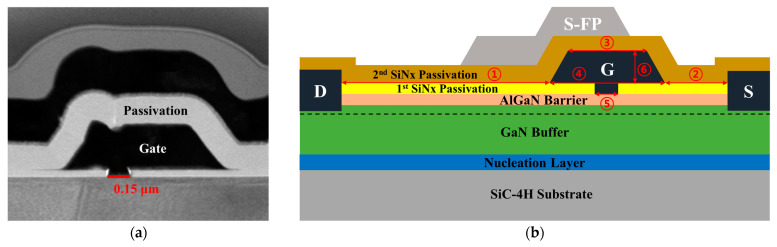
A cross-sectional schematic of the fabricated planar gate AlGaN/GaN high-electron-mobility transistor (HEMT) structure: (**a**) scanning electron microscope (SEM) image; and (**b**) an illustration used in modeling. The S, D, G, and S-FP represent the source, drain, gate, and source-connected field-plate, respectively; each number (1–6) is explained in Table 1.

To obtain a reasonable simulation criterion, we first analyzed the fabricated HEMT with a 0.15-μm planar-gate structure [18]. The AlGaN/GaN HEMTs were grown on a 4-inch SiC substrate by using metal–organic chemical vapor deposition. More precisely, the epitaxial layers were composed of a 0.2-µm-thick nucleation layer, a Fe-doped 2-µm-thick GaN buffer layer, and a 25-nm-thick Al_0.25_Ga_0.75_N barrier layer. Additionally, the Ohmic metallization of the device was formed by Ti/Al/Ni/Au evaporation followed by rapid thermal annealing at 775 °C for 30 s, and device isolation was achieved by P^+^ ion implantation. Next, a 50-nm-thick 1st Si_3_N_4_ passivation layer was deposited by using plasma-enhanced chemical vapor deposition (PECVD). The first metal interconnections with the Ohmic contacts were formed by the Ti/Au evaporation after etching the 1st Si_3_N_4_ passivation layer. Further, a planar gate was formed by using single-layer electron beam lithography. More precisely, a gate foot length of 0.15 µm was obtained by electron-beam exposure using poly methyl methacrylate resist, and the 1st Si_3_N_4_ passivation layer underneath the gate pattern was removed by inductively coupled plasma dry etching. Ni/Au planar-gate metal stack was deposited by electron-beam evaporation and lift-off processes. After this, a 250-nm-thick 2nd Si_3_N_4_ PECVD film was deposited for device passivation. A source-connected field-plate was formed by using the Ti/Au metal and lift-off process. Finally, the wafer-thinning and backside via-hole process was performed. The scanning electron microscope (SEM) image of the fabricated planar gate AlGaN/GaN HEMT is shown in Figure 1a.

Figure 1b shows the schematic diagram of the basic Si_3_N_4_ passivation structure of the HEMT. Based on the fabricated device, we determined the structural and material parameters to be utilized for modeling without any other structural changes, such as changes to the planar-gate electrode structure, and while retaining the same gate foot-length of 0.15 μm, including the epitaxial layer. Table 1 provides the specific geometrical parameter information of the basic Si_3_N_4_ passivation structure used in the simulation.

In this simulation study, it is essential to initialize the material and simulation parameters in order to accurately confirm the operating characteristics of the device. The specific material parameters of GaN and AlGaN used for simulation are summarized in Table 2. As shown in Table 2, we subdivided the FMCT (Farahmand-modified Caughey–Thomas) and GANSAT electron mobility models based on the electric field within the device [19]. Additionally, heat models were applied in the simulation to implement the actual device performance for accurate simulation results. Additionally, acceptor-trap doping in AlGaN/GaN HEMTs is generally used to improve the $V_{BD}$ by reducing the substrate leakage current [20]. However, current-collapse phenomena such as drain-lag and gate-lag inevitably occur [21]. Therefore, a properly controlled acceptor-trap doping is essential to achieve high-performance HEMTs. The Gaussian acceptor doping profile is applied in the simulation by using Fe (iron). More precisely, the peak acceptor-trap doping concentration is set to 10^18^/cm^2^ in the GaN buffer layer and gradually decreases according to the Gaussian distribution, resulting in an acceptor-trap doping concentration below 10^15^/cm^2^ at the interface between AlGaN and GaN.

In order to conduct an accurate device simulation by considering the self-heating effect (SHE), we applied physical models to calculate the heat generation within the device [22,23]. First, we used the lattice heat flow equation,

(1) $$C\frac{\partial T_{L}}{\partial t}=\nabla \left(\kappa \nabla T_{L}\right)+H$$

where C is the heat capacitance per unit volume, κ is the thermal conductivity coefficient, H is the heat generation, and $T_{L}$ is the local lattice temperature. More precisely, the thermal conductivity, which is important to calculate the SHE in a device simulation, is commonly temperature-dependent. Therefore, we applied the thermal conductivity model,

(2) $$\kappa \left(T\right)=(TC.CONST)/{\left(T_{L}/300\right)}^{TC.NPOW}$$

where TC.CONST is the thermal conductivity constant at 300 K and TC.NPOW is the calibration factor which is an experimental value. The applied TC.CONST and TC.NPOW parameters of GaN, AlGaN, and SiC-4H are summarized in the Table 3 [24].

We investigated the relationship between parasitic capacitances and frequency characteristics. The capacitance equation can be expressed by:

(3) $$C=\frac{\varepsilon_{o}\varepsilon_{r}}{d}A$$

where C is the capacitance, $\varepsilon_{o}$ is the permittivity of free space (constant value), $\varepsilon_{r}$ is the dielectric constant of the material, A is the area of overlap of the two electrodes, and d is the electrode separation distance. As expressed in Equation (3), $\varepsilon_{r}$ and d have a significant influence on the change in capacitance.

Next, $f_{T}$ and maximum oscillation frequency ($f_{max}$) were explained by Equations (4) and (5):

(4) $$f_{T}=\frac{g_{m}}{2\pi \left(C_{gs}+C_{gd}\right)}\approx \frac{g_{m}}{2\pi C_{gs}}$$

(5) $$f_{max}=\frac{f_{T}}{2\sqrt{\pi f_{T}C_{gd}(R_{s}+R_{g}+R_{gs}+{2\pi L}_{s})+G_{ds}\left(R_{s}+R_{g}+R_{gs}+\pi f_{T}L_{s}\right)}}\approx \sqrt{\frac{f_{T}}{8\pi R_{g}C_{gd}}}$$

where $g_{m}$, $C_{gs}$, and $C_{gd}$ represent the transconductance, gate-to-source capacitance, and gate-to-drain capacitance, respectively. As described in Equation (4), decreasing the parasitic capacitances, such as $C_{gs}$ and $C_{gd}$, increases the $f_{T}$. The $R_{s}$, $R_{g}$, $R_{gs}$, $L_{s}$, and $G_{ds}$ are the source resistance, gate resistance, gate-to-source resistance, parasitic source inductance, and output conductance, respectively [25]. Equation (5) shows that $R_{g}$ and $C_{gd}$ must be reduced to achieve a higher $f_{max}$. Additionally, as $f_{T}$ increases, $f_{max}$ also increases, as shown in Equation (5).

## 3. Results

### 3.1. Basic Si_3_N_4_ Passivation Structure of HEMT Modeling Verified by Matching the Simulation’s Results with the Measured Data

**Figure 2:**
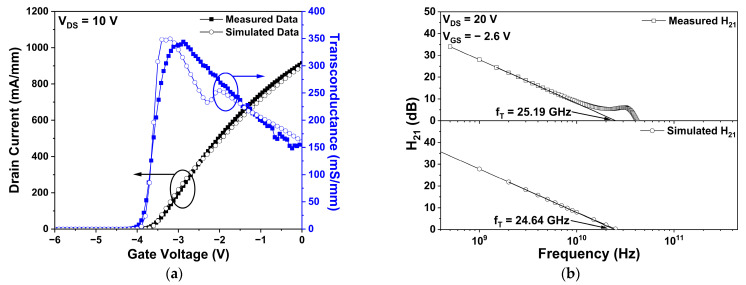
(**a**) The measured and simulated drain current-gate voltage ($I_{DS}–V_{GS}$) transfer characteristics of a basic Si_3_N_4_ passivation structure of the HEMT at a drain voltage ($V_{DS}$ ) of 10 V. The black and blue arrows represent drain current and transconductance, respectively; and (**b**) the measured and simulated current gain of a basic Si_3_N_4_ passivation structure of the HEMT as a function of frequency at $V_{DS}$ = 20 V and gate voltage ($V_{GS}$ ) = −2.6 V.

In this work, we matched the simulated drain current-gate voltage ($I_{DS}$-$V_{GS}$) transfer and $f_{T}$ with the measured data of the fabricated basic Si_3_N_4_ passivation structure of the HEMT to ensure the simulation’s reliability. The measured datum of the drain current at a gate voltage of 0 V ($I_{dss}$) was 898.71 mA/mm, which was similar to the simulated datum of 914.90 mA/mm. Furthermore, the measured maximum transconductance ($G_{m}$) was 344.17 mS/mm, which corresponds to the simulated value of 349.60 mS/mm. The above results for maintaining the threshold voltage ($V_{th}$) at −3.8 V were confirmed. Therefore, by adjusting the simulation’s parameters, $I_{dss}$, $G_{m}$, and $V_{th}$ values of the simulation and the corresponding measured results were matched within 1.8% of the maximum error rate, as shown in Figure 2a. A dip of the simulated transconductance around-gate voltage of −2.4 V was found, since two different field-dependent electron mobility models were used, as represented in Table 2. The exact criterion for determining the field within the device as low or high remains unknown, but a slight dip in simulated transconductance can occur at an obscure boundary of these models. The $I_{DS}$–$V_{GS}$ transfer of the fabricated device was measured by using a Cascade Microtech Summit 12,000 probe station and a HP4142B Modular DC Source/Monitor probe station.

The simulated and measured $f_{T}$ of the basic Si_3_N_4_ passivation structure of HEMT are shown in Figure 2b. As regards the RF characteristics, the bias points of the simulated results and the measured data were a drain voltage of 20 V and gate voltage of −2.6 V, which were selected since the frequency characteristics were outstanding in comparison to other bias points. More specifically, the $f_{T}$ was defined as the intersection of the x-axis and the extension line at the point of current gain (H_21_), with a slope of −20 dB/decade [26]. The measured and simulated values of the $f_{T}$ were 25.19 GHz and 24.64 GHz, respectively. This clearly shows that the simulated $f_{T}$ was accurate enough when compared to the measured values, as the error rate was only 2.2%. PNA-X N5245A network analyzer was used to analyze the $f_{T}$ of the device within the frequency range from 0.5 to 50 GHz.

### 3.2. Comparative Analysis between HEMTs with Si_3_N_4_, HfO_2_, and Hybrid Passivation Structures

**Figure 3:**
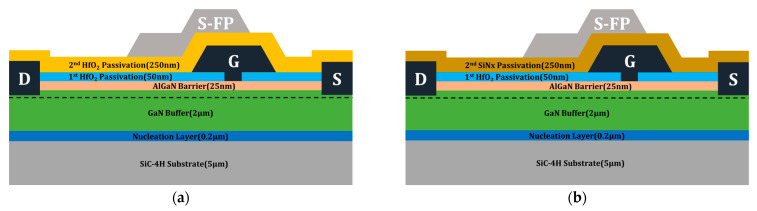
The schematics of various passivation structures for the AlGaN/GaN HEMT: (**a**) HfO_2_ passivation structure; and (**b**) hybrid passivation structure.

To enhance the operational characteristics, we suggested two structures, as shown in Figure 3. Figure 3a shows the HfO_2_ passivation structure of the HEMT. As shown in Figure 3b, the hybrid passivation structure consists of first and second passivation layers, which are composed of HfO_2_ and Si_3_N_4_, respectively. Specifically, these passivation structures will exhibit enhanced DC characteristics, including the $V_{BD}$, as compared to the basic Si_3_N_4_ passivation structure, because of the material properties of HfO_2_. The other structural parameters excluding the passivation material were not changed in the simulation.

#### 3.2.1. Analysis of DC Characteristics

**Figure 4:**
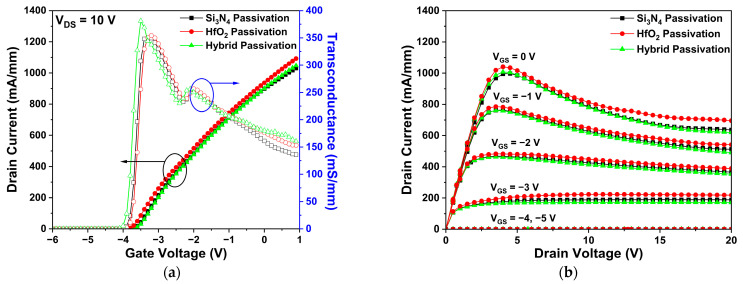
The DC simulation results of Si_3_N_4_, HfO_2_, and hybrid passivation structures: (**a**) $I_{DS}–V_{GS}$ transfer at $V_{DS}$ = 10 V. The black and blue arrows represent drain current and transconductance, respectively; (**b**) drain current-drain voltage ($I_{DS}$ -$V_{DS}$ ) characteristics at $V_{GS}$ = −5, −4, −3, −2, −1, and 0 V.

First, we analyzed the DC characteristics of the HfO_2_ and hybrid passivation-structures, and then compared them to the basic Si_3_N_4_ passivation-structure. Figure 4a shows the $I_{DS}–V_{GS}$ transfer characteristics of all three structures at a drain voltage of 10 V. Among them, the HfO_2_ passivation structure slightly improved not only the drain current, but also the transconductance, in comparison with the basic Si_3_N_4_ passivation structure. Interestingly, these results show that $R_{on}$ decreases as HfO_2_ is employed in passivation [27]. The drain current-drain voltage ($I_{DS}–V_{DS}$) characteristics were simulated at the gate voltages of −5, −4, −3, −2, −1, and 0 V, respectively, as shown in Figure 4b. As the higher gate voltage was applied, the electron concentration in the channel region increased, resulting in a large drain current. However, a decrease in drain current was observed as the drain voltage increased. These results may be explained by SHE, since applying a higher voltage leads to a higher heat generation, resulting in the degradation of the DC characteristics [28,29,30]. When the applied drain voltage increased, a strong electric field was generated within the device. Due to the large electric field, phonon scattering was observed to reduce the electron mobility and current density. Although the SHE occurred in all three structures, the HfO_2_ passivation and hybrid passivation structures exhibited a higher drain current than did the basic Si_3_N_4_ passivation structure. In addition, $R_{on}$ was calculated to be 4.02, 3.84, and 3.97 Ω-mm for the basic Si_3_N_4_, HfO_2_, and hybrid passivation structures, respectively.

**Figure 5:**
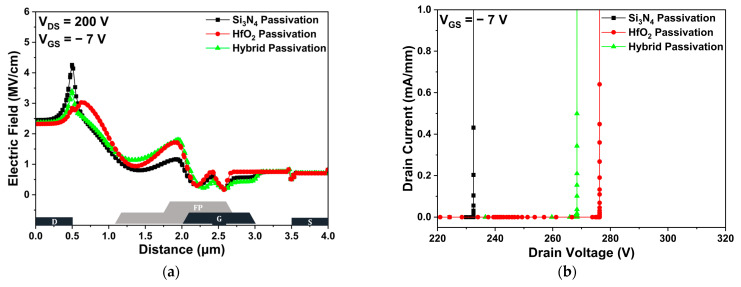

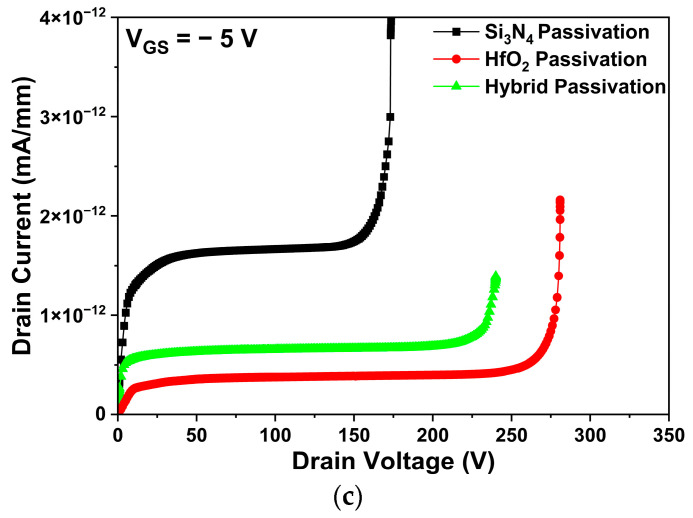
The DC simulation results of Si_3_N_4_, HfO_2_, and hybrid passivation structures: (**a**) electric field distribution in the channel region; (**b**) off-state breakdown voltage; and (**c**) off-state drain leakage current.

Figure 5a shows the electric field distribution in the channel layer under a drain voltage of 200 V. In comparison with the basic Si_3_N_4_ passivation structure, the HfO_2_ and hybrid passivation structures demonstrated that the peak electric field in the channel layer was reduced and dispersed due to the high dielectric constant of HfO_2_. As the peak electric field increased, impact ionization, which causes the generation of electron-hole pairs, became severe. Thus, the redistribution of the electric field effectively improved the V_BD_. Specifically, the $V_{BD}$ values of the Si_3_N_4_, HfO_2_, and hybrid passivation structures were 232.47, 276.27, and 268.41 V, respectively, as shown in Figure 5b. After applying a voltage of −7 V to the gate to completely turn off the device, the drain voltage when the drain current exceeded 1 mA/mm was defined as the $V_{BD}$. Figure 5c compares the drain leakage current for the three different passivation structures. Particularly, the structures where HfO_2_ is applied to the passivation layer can show that the 2-DEG confinement in the channel region can be improved due to the wide bandgap energy of HfO_2_, reducing the leakage current. Therefore, the HfO_2_ passivation structure exhibited the least drain leakage current among the three [31,32].

#### 3.2.2. Analysis of the RF Characteristics

**Figure 6:**
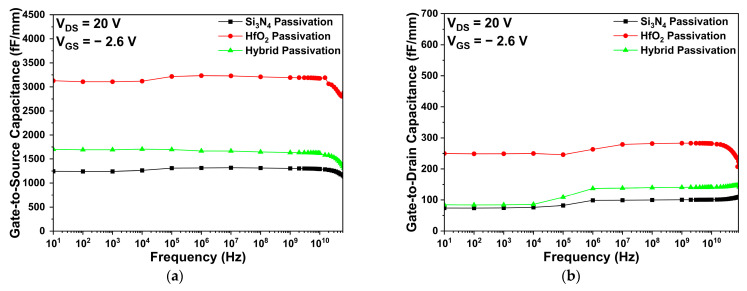
The parasitic capacitance characteristics of Si_3_N_4_, HfO_2_, and hybrid passivation structures: (**a**) gate-to-source capacitance; and (**b**) gate-to-drain capacitance.

Figure 6 shows the parasitic capacitance characteristics for Si_3_N_4_, HfO_2_, and hybrid passivation structures. Specifically, the $C_{gs}$ and $C_{gd}$ were obtained at a drain voltage of 20 V and a gate voltage of −2.6 V. As shown in Figure 6a,b, the HfO_2_ passivation structure shows the highest $C_{gs}$ and $C_{gd}$, since the dielectric constant of HfO_2_ is larger than that of Si_3_N_4_, which is explained by Equation (3). In addition, the parasitic capacitance values of the hybrid passivation structure were smaller than that of the HfO_2_ passivation structure. This is because the HfO_2_ passivation thickness was thinner in the hybrid passivation structure compared to the HfO_2_ passivation structure. Therefore, the parasitic capacitances tended to increase as more HfO_2_ was used in the passivation layer.

**Figure 7:**
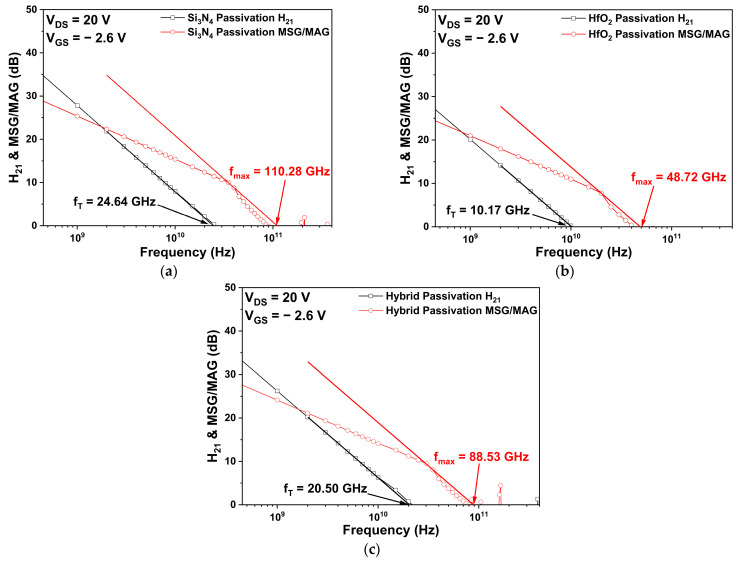
The cut-off frequency ($f_{T}$) and maximum oscillation frequency ($f_{max}$ ) for different passivation structures: (**a**) Si_3_N_4_ passivation structure; (**b**) HfO_2_ passivation structure; and (**c**) hybrid passivation structure.

Figure 7 represents the simulated $f_{T}$ and $f_{max}$ of the three different passivation structures. Similarly, as the capacitance simulations, $f_{T}$ and $f_{max}$, were obtained at a drain voltage of 20 V and a gate voltage of −2.6 V. More precisely, the $f_{T}$ values are 24.64, 10.17, and 20.50 GHz for the basic Si_3_N_4_ passivation, HfO_2_ passivation, and hybrid passivation structures, respectively. The $f_{T}$ values of the HfO_2_ and hybrid passivation structures were decreased by 58.7% and 16.8% compared to the basic Si_3_N_4_ passivation structure, respectively. According to Equation (4), the $f_{T}$ values of the three passivation structures may have been influenced by the $g_{m}$ and $C_{gs}$. In addition, the $f_{max}$ values of the basic Si_3_N_4_ passivation, HfO_2_ passivation, and hybrid passivation structures are 110.28, 48.72, and 88.53 GHz, respectively. It can be seen that $f_{max}$ value of HfO_2_ passivation structure significantly decreased as $f_{T}$ decreased according to Equation (5). Particularly, the $f_{max}$, which is obtained from the extension line with a slope of −20 dB/decade at the intersection of the maximum stable/available gain (MSG/MAG), becomes 0 dB [33,34].

Interestingly, these results clearly show that the ratio of HfO_2_ in passivation is important for DC and RF performances. As the ratio of HfO_2_ increases, the DC characteristics are improved, but the RF characteristics, such as parasitic capacitances and frequency characteristics, are degraded due to the material properties of HfO_2_. To improve both DC and RF characteristics, we selected the hybrid passivation structure and then simulated four different 2nd Si_3_N_4_ passivation thicknesses, i.e., 150, 250, 350, and 450 nm, which will be discussed in Section 3.3. More precisely, to optimize the second Si_3_N_4_ passivation thickness and calculate the figure-of-merit, we analyzed the operational characteristics including $V_{BD}$, parasitic capacitances, and frequency characteristics.

### 3.3. Determination of the Optimum Second Passivation Thickness for Hybrid Structure

#### 3.3.1. Analysis of the DC Characteristics

**Figure 8:**
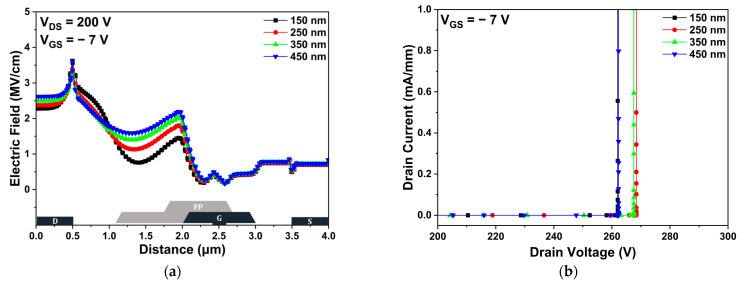
The DC simulation results of hybrid passivation structure with various second passivation thicknesses: (**a**) electric field distribution in the channel region; and (**b**) off-state breakdown voltage.

Figure 8a shows the electric field distribution in the channel region at a drain voltage of 200 V and a gate voltage of −7 V. The peak electric field was not significantly affected by the second passivation thickness. Additionally, the overall electric field distribution also showed no significant difference. Therefore, the $V_{BD}$ values of the various second passivation thickness structures were not changed significantly. As shown in Figure 8b, the $V_{BD}$ was simulated to be 262.00, 268.41, 267.57, and 262.30 V for the hybrid passivation structure with second passivation thicknesses of 150, 250, 350, and 450 nm, respectively. As the field-plate gradually deviates from the channel region, the electric field in the channel cannot be dispersed, resulting in the decrease of $V_{BD}$. Meanwhile, as the passivation thickness increases, it is expected that $V_{BD}$ would increase, because the passivation can prevent the electric field in the channel region spread by the high electric field adjacent to the gate electrode. For these two reasons, the $V_{BD}$ were slightly enhanced in the second passivation thicknesses of 250 and 350 nm, compared with other structures.

#### 3.3.2. Analysis of the RF Characteristics

**Figure 9:**
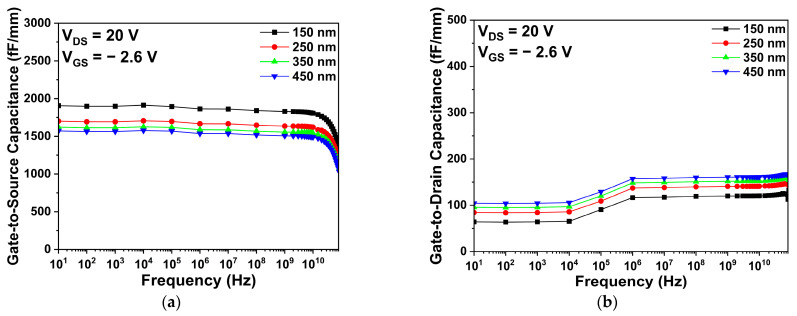
The parasitic capacitance characteristics of the hybrid passivation structure with various second passivation thicknesses: (**a**) gate-to-source capacitance; and (**b**) gate-to-drain capacitance.

Figure 9 shows the $C_{gs}$ and $C_{gd}$ of the hybrid passivation structure with various second passivation thicknesses, at a drain voltage of 20 V and a gate voltage of −2.6 V. The second passivation thickness affected the parasitic capacitance values. Specifically, the 150-nm-thick second passivation structure showed the largest $C_{gs}$, due to the decrease in distance between the gate and source, as shown in Figure 9a. According to Equation (3), as the distance among the electrodes increased, the parasitic capacitances decreased. Therefore, compared to $C_{gs}$, there is no significant change in $C_{gd}$, because the gate-to-source distance is much shorter than the gate-to-drain distance. In addition, the 450-nm-thick second passivation structure exhibited a slightly larger $C_{gd}$ than did the other structures, as shown in Figure 9b. The change in materials from air to Si_3_N_4_ led to an increase in $C_{gd}$ due to dielectric constant of the materials, which is explained by Equation (3).

**Figure 10:**
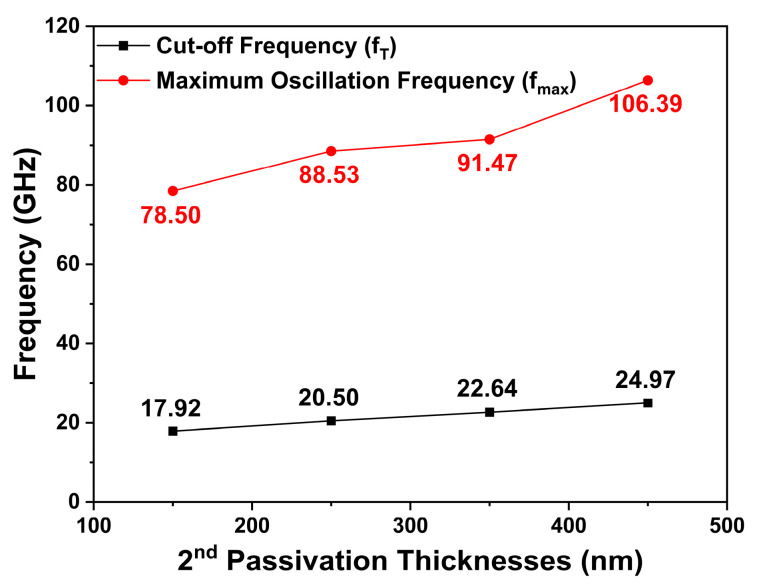
The simulated $f_{T}$ and $f_{max}$ as a function of the second passivation thicknesses at $V_{DS}$ = 20 V and $V_{GS}$ = −2.6 V.

Figure 10 shows the simulated $f_{T}$ and $f_{max}$ values for the different second passivation thicknesses at a drain voltage of 20 V and a gate voltage of −2.6 V. When the second passivation thicknesses were 150, 250, 350, and 450 nm, the $f_{T}$ values in the simulations were 17.92, 20.50, 22.64, and 24.97 GHz, respectively. A decrease in the $C_{gs}$ due to a change in the second passivation thickness led to an increase in $f_{T}$, according to Equation (4). Therefore, $f_{T}$ tended to increase by about 14.4~39.3% as the second passivation thickness was extended by each 100-nm-step. The $f_{max}$ values were simulated to be 78.50, 88.53, 91.47, and 106.39 GHz for the hybrid passivation structure with the second passivation thicknesses of 150, 250, 350, and 450 nm, respectively. Comparing the $f_{max}$ values of the hybrid passivation structures based on the different second passivation thicknesses, it can be demonstrated that the $f_{max}$ values increased by 12.8~35.5% with each 100-nm-step increase in the second passivation thickness. According to Equation (5), the $f_{max}$ values were mainly influenced by the increase in $f_{T}$ because there was no significant change in $C_{gd}$. Throughout these results, we confirmed the dependence of frequency characteristics in relation to the second passivation thickness.

## 4. Discussion

In this article, we simulated the DC and RF characteristics of various passivation structures. Additionally, we analyzed the hybrid passivation structure by changing the second passivation thickness. Based on these results, we first calculated the LFOM and JFOM to investigate the performance of the device for the various passivation structures. Table 4 provides a summary of the DC and RF characteristics, including the figure-of-merit for the four different passivation structures. More precisely, the LFOM and JFOM of the basic Si_3_N_4_ passivation structures were 13.44 MW/mm and 5.73 THz-V, respectively. The HfO_2_ passivation structure increased the LFOM by 48% and decreased the JFOM by 39% compared with the basic Si_3_N_4_ passivation structure. In comparison with the basic Si_3_N_4_ passivation structure, analysis of the hybrid passivation structure showed that the LFOM was increased by up to 35% and the JFOM was decreased by up to 4%.

Subsequently, the LFOM values for the hybrid passivation structure of different second passivation thicknesses were estimated to be 17.93, 18.15, 17.68, and 15.53 MW/mm, respectively. In addition, except for the hybrid passivation structure with 450-nm-thick second Si_3_N_4_ passivation, the LFOM values of the other hybrid passivation structures had improved by more than 28%, compared to the basic Si_3_N_4_ passivation structure. Further, we measured the JFOM values for the hybrid passivation structures of different second passivation thicknesses, which were 4.70, 5.50, 6.06, and 6.55 THz-V, respectively. As the second passivation thickness increased, the JFOM values also increased.

## 5. Conclusions

In this study, using TCAD simulation, we analyzed the operational characteristics of AlGaN/GaN HEMTs in accordance with changes of passivation materials and thicknesses. Before analyzing the various passivation structures, all the simulation and material parameters were precisely set through mapping with the measurement data of the fabricated device to ensure the reliability of the simulated data. Based on the simulation results, we suggest an optimized hybrid structure of HEMT which adopts a 50-nm-thick first HfO_2_ passivation and a 350-nm-thick second Si_3_N_4_ passivation. Unlike other general structures such as the field-plate in the HEMT, we confirmed that the hybrid passivation structure of the HEMT with suitable passivation thickness could enhance both the DC and RF performances, including the LFOM and JFOM. Consequently, the simulation results clearly show that HfO_2_ as a passivation material with a second passivation thickness suitable for the AlGaN/GaN HEMTs can be a promising candidate for future high-power and high-frequency applications.

## Figures and Tables

**Table 1:** The geometric parameters of the basic Si_3_N_4_ passivation structure of HEMT.

Parameters	Value (μm)
① $L_{Gate-Drain}$	1.5
② $L_{Gate-Source}$	0.5
③ $L_{Gate-Head\;top}$	0.8
④ $L_{Gate-Head\;bottom}$	1.0
⑤ $L_{Gate-Foot}$	0.15
⑥ $L_{Gate-Height}$	0.44
Field-plate thickness	0.44
1st passivation	0.05
2nd passivation	0.25
AlGaN barrier	0.025
GaN buffer	2
Nucleation layer	0.2

**Table 2:** Material parameters used in the simulation at a temperature of 300 K (SRH: Shockley–Read–Hall).

Parameters	Units	GaN	AlGaN
Bandgap energy	eV	3.39	3.88
Electron affinity	eV	4.2	2.3
Relative permittivity	-	9.5	9.38
Low field mobility model	-	FMCT Mobility Model	
High field mobility model	-	GANSAT Mobility Model	
Electron saturation velocity	cm/s	1.9 ×10^7^	1.12 ×10^7^
Hole saturation velocity	cm/s	1.9 ×10^7^	1.00 ×10^6^
Electron SRH lifetime	s	1.0 ×10^–8^	1.0 ×10^–8^
Hole SRH lifetime	s	1.0 ×10^–8^	1.0 ×10^–8^

**Table 3:** Thermal parameters used for the thermal conductivity model.

Parameters	Units	GaN	AlGaN	SiC-4H
TC.CONST	-	1.3	0.4	3.3
TC.NPOW	-	0.43	0	1.61

**Table 4:** A summary of the DC and RF characteristics of various passivation structure HEMTs.

Parameters	Units	Si_3_N_4_	HfO_2_	Hybrid	
First/second passivation thickness	nm	50/250	50/250	50/250	50/350
$\mathrm{On}\text{-}\mathrm{resistance}\;(R_{on}$)	Ω-mm	4.02	3.84	3.97	4.16
$\mathrm{Breakdown}\;\mathrm{voltage}\;(V_{BD}$)	V	232.47	276.27	268.41	267.57
$\mathrm{Cut}\text{-}\mathrm{off}\;\mathrm{frequency}\;(f_{T}$)	GHz	24.64	10.17	20.50	22.64
$\mathrm{Maximum}\;\mathrm{oscillation}\;\mathrm{frequency}\;(f_{max}$)	GHz	110.27	48.72	88.53	91.47
Standard lateral figure-of-merit (LFOM)	MW/mm	13.44	19.93	18.15	17.21
Johnson’s figure-of-merit (JFOM)	THz-V	5.73	2.81	5.50	6.06
